# Hamman-Rich Syndrome: A Diagnosis of Exclusion in the COVID-19 Pandemic

**DOI:** 10.7759/cureus.9866

**Published:** 2020-08-19

**Authors:** Qian Zhang, Ahmad Raza, Vincent Chan, Artem Minalyan, John Madara

**Affiliations:** 1 Internal Medicine, Abington Hospital - Jefferson Health, Abington, USA; 2 Internal Medicine, Abington Hospital- Jefferson Health, Abington, USA; 3 Pulmonary and Critical Care, Abington Hospital- Jefferson Health, Abington, USA

**Keywords:** acute interstitial pneumonia, hamman-rich syndrome, idiopathic interstitial pneumonia, acute respiratory distress syndrome [ards]

## Abstract

Hamman-Rich syndrome is a rapidly progressive interstitial lung disease with acute respiratory distress syndrome physiology. It carries a grave prognosis and a high early mortality rate. It is often distinguished from other similar pulmonary pathologies based on the clinical course, laboratory findings, bronchoalveolar lavage testing, and pathology report. We detail a 77-year-old lady with no prior pulmonary disease, smoking history, or occupational and environmental exposures present to the emergency department found to be in acute hypoxic respiratory failure with impressive progressive radiographic findings. The presumptive diagnosis of Hamman-Rich syndrome was made based on a combination of factors after ruling out other similar clinical entities, especially in the setting of an ongoing COVID-19 pandemic.

## Introduction

Hamman-Rich syndrome, or acute interstitial pneumonia (AIP), is a rare idiopathic pulmonary disease that leads to fulminant respiratory failure [1]. It was first discovered by Louis Hamman and Arnold Rich in 1935 [2]. AIP's clinical presentation, radiological, and physiological findings are similar to that of acute respiratory distress syndrome (ARDS). It is characterized clinically by its rapid onset of respiratory failure as it could often be confused with other clinical entities of interstitial pneumonia. Unfortunately, it has an extremely poor prognosis with >70% three-month mortality rate despite aggressive medical management [3].

## Case presentation

**Figure 1:**
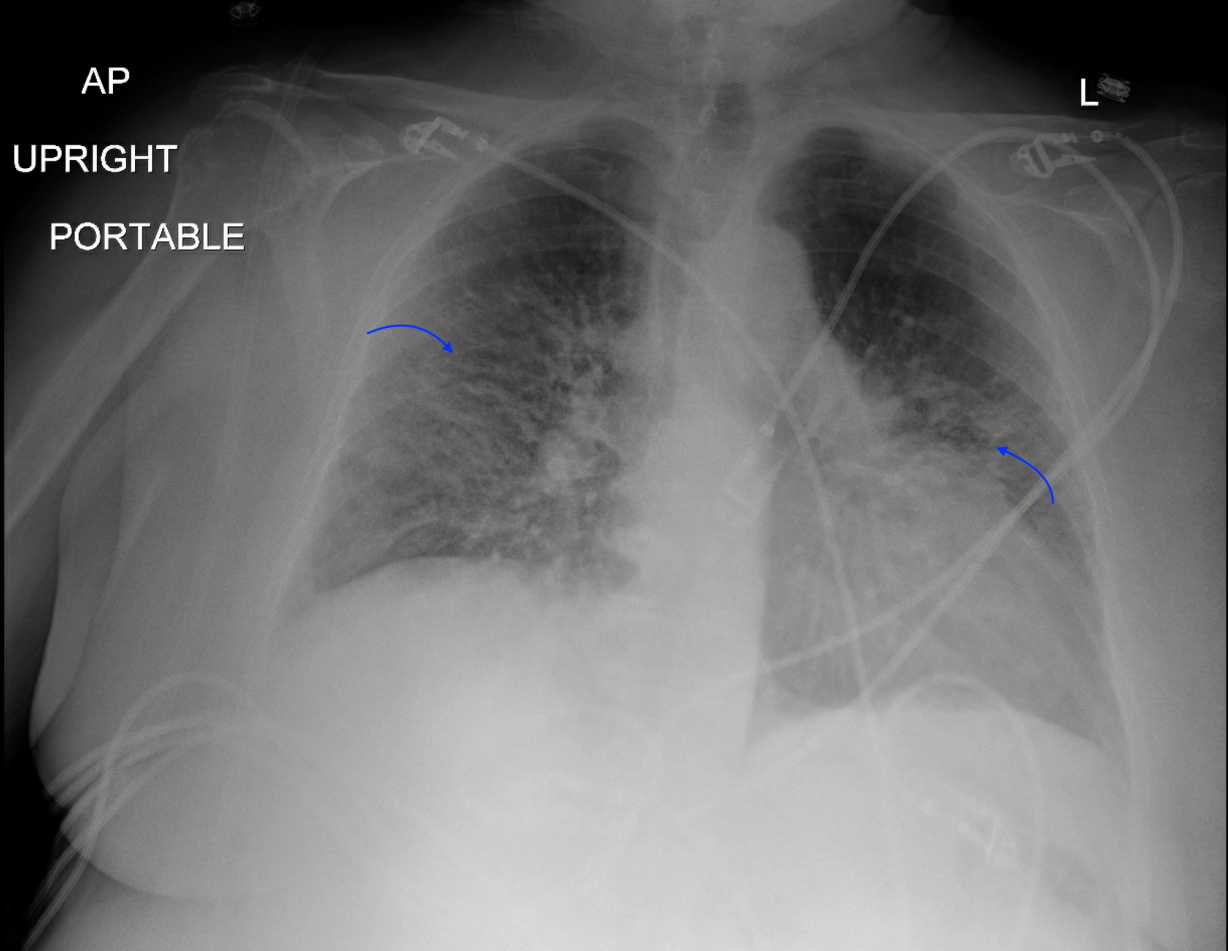
Chest X-Ray Bilateral patchy asymmetrical opacities without cardiomegaly or pleural effusions. AP: anterior-posterior; L: left.

**Figure 2:**
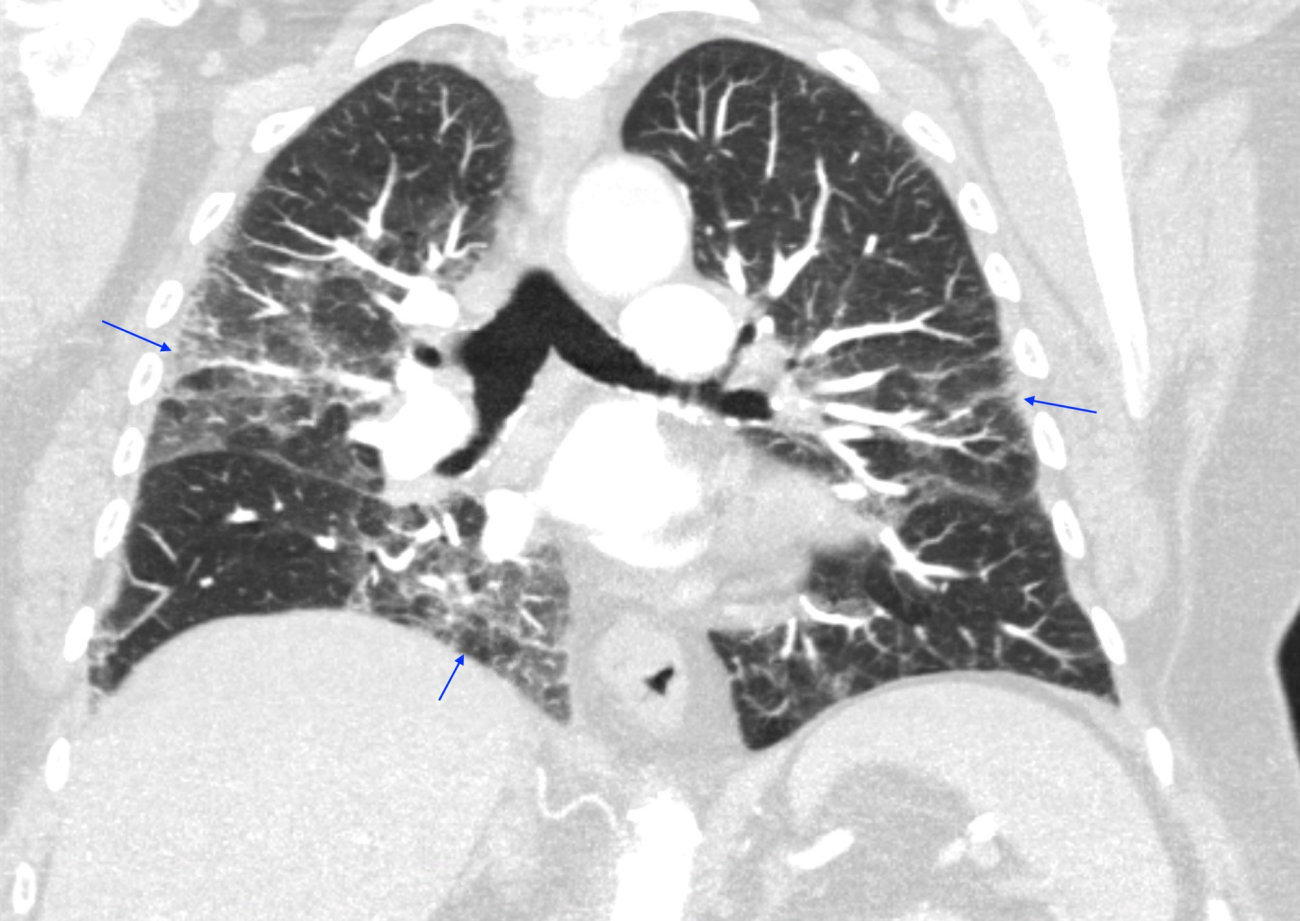
CT Chest (Coronal View) Bilateral patchy ground-glass opacities without intraluminal defects, cardiomegaly, or pleural effusions.

Our patient was a 77-year-old lady who presented to the emergency department (ED) with the chief complaint of shortness of breath. Her past medical history was significant for coronary artery disease (CAD) status post stent placement, moderate aortic stenosis, depression, and anxiety. She denied a family history of cardiac or pulmonary conditions. She was fully functional independently at baseline, not on home oxygen, and denied the use of tobacco, alcohol, or illicit medications. There were no reported recent travel history or sick contacts. She worked as a florist for many years but denied other occupational or environmental exposures. Her outpatient medications included Aspirin 81 mg PO daily, clopidogrel 75 mg PO daily, and fluoxetine 40 mg PO daily. She reported a viral-like illness approximately two weeks ago with the symptoms of intermittent low-grade fever, non-productive cough, and shortness of breath. Her primary care physician prescribed albuterol and methylprednisolone without symptomatic relief and thus she decided to present to the ED for further evaluation. Initial vital signs on ED presentation: temperature max 99.8°F, blood pressure 117/62 mmHg, heart rate 80 beats per minute, respiratory rate 18 breaths per minute, and pulse oximetry saturation at 97% on 3 liters nasal cannula (NC). Physical examination was overall benign except for decreased bibasilar breath sounds. Review of systems was negative for chest pain, orthopnea, paroxysmal nocturnal dyspnea, extremity edema, weight changes, sore throat, chills, night sweat, or bowel habit changes. Initial laboratory findings were significant for serum sodium 135 mEq/L, cardiac troponin <0.10 ng/mL, creatinine 0.69 mg/dL, white blood cell count 8.9 K/uL, hemoglobin 14.7 g/dL, absolute lymphocyte count 1.0 K/uL, d-dimer plasma 433 ng/mL DDU, sedimentation rate 54 mm/h, ferritin 159 ng/mL, C-reactive protein 93.26 mg/L, and negative Abbott RealTime SARS-CoV-2 assay. Chest X-ray (CXR) showed bilateral patchy asymmetrical opacities without cardiomegaly or pleural effusions (Figure 1). Computerized tomography (CT) scan of the chest with contrast revealed bilateral patchy ground-glass opacities without intraluminal defects, cardiomegaly, or pleural effusions (Figure 2). Hologic SARS-CoV-2 assay was sent while the patient was placed in airborne isolation. No antibiotics were administered as there was a low clinical suspicion of bacterial pneumonia.

On days 2-3 of hospitalization, the patient remained afebrile and hemodynamically stable, maintained oxygen saturation above 90% on 3L NC. The most likely differential diagnosis was COVID-19 pneumonitis despite the initial negative test result in the setting of the COVID-19 pandemic. She was treated with methylprednisolone 40 mg b.i.d. while trending the daily inflammatory markers. Blood cultures, sputum cultures, urine legionella, and nasal swab Methicillin-resistant Staphylococcus aureus (MRSA) were sent. She was placed on prophylactic deep venous thrombosis (DVT) regimen and maintained on enhanced respiratory precautions.

On day 4 of hospitalization, she had worsening of respiratory status and she was placed on a non-rebreather (NRB) in order to maintain oxygen saturation above 90%. She was subsequently upgraded to the medical intensive care unit (MICU) due to worsening hypoxic respiratory failure. The clinical assessment of the patient at this point was believed to be an atypical presentation of COVID-19 pneumonitis despite two negative COVID-19 assays, mild elevation of the inflammatory markers without up-trending, and a lack of significant vital signs instability to support cytokine storm. Thus, it was determined to defer the use of remdesivir and tocilizumab but rather increased methylprednisolone 40 mg to t.i.d. There was a low clinical suspicion of a cardiogenic component of acute hypoxic respiratory failure as the cardiac B-type natriuretic peptide (BNP) was 85 pg/mL. Culture data were also negative.

**Figure 3:**
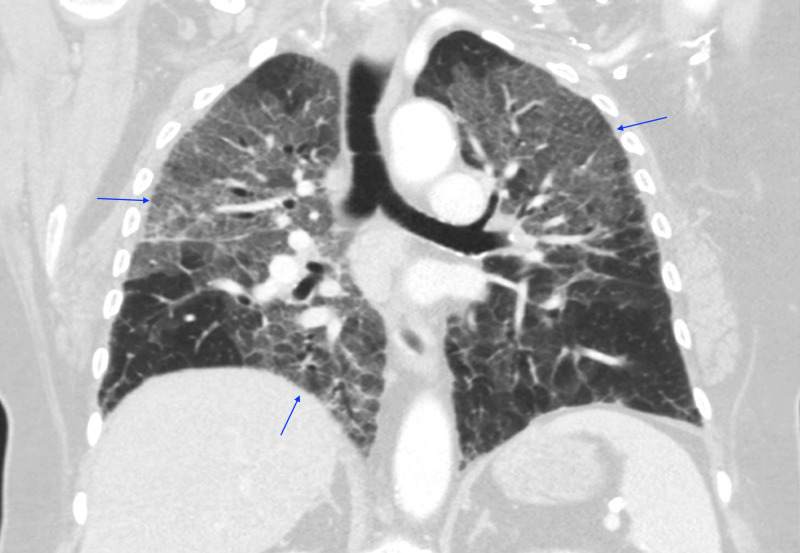
CT Chest (Coronal View) Multifocal ground-glass opacities predominately affecting the right lung in addition to the worsening of the left apical lobe comparing to the previous study.

On days 5-13 of hospitalization, her oxygen requirement gradually improved with the treatment of daily methylprednisolone, albuterol p.r.n., and low dose intravenous furosemide p.r.n. to keep a negative fluid balance. Bactrim was added to her medical regimen for pneumocystis pneumonia (PCP) prophylaxis in the setting of steroid treatment. The enhanced respiratory precaution was discontinued as there were three negative COVID-19 assays. Echocardiogram did not reveal cardiomyopathy or evidence of pulmonary hypertension. A repeat CT chest with contrast revealed multifocal ground-glass opacities predominately affecting the right lung in addition to the worsening of the left apical lobe comparing to the previous study (Figure 3). A detailed autoimmune panel including anti-cyclic citrullinated peptide (anti-CCP), cardiolipin antibodies, anti-Jo-1 antibody, antinuclear antibody (ANA), and anti-neutrophil cytoplasmic antibody (ANCA) was sent. She was subsequently downgraded from MICU and was discharged from the hospital while on 4L NC. She was instructed to continue steroids tapering and follow up with the pulmonologist in the outpatient setting.

**Figure 4:**
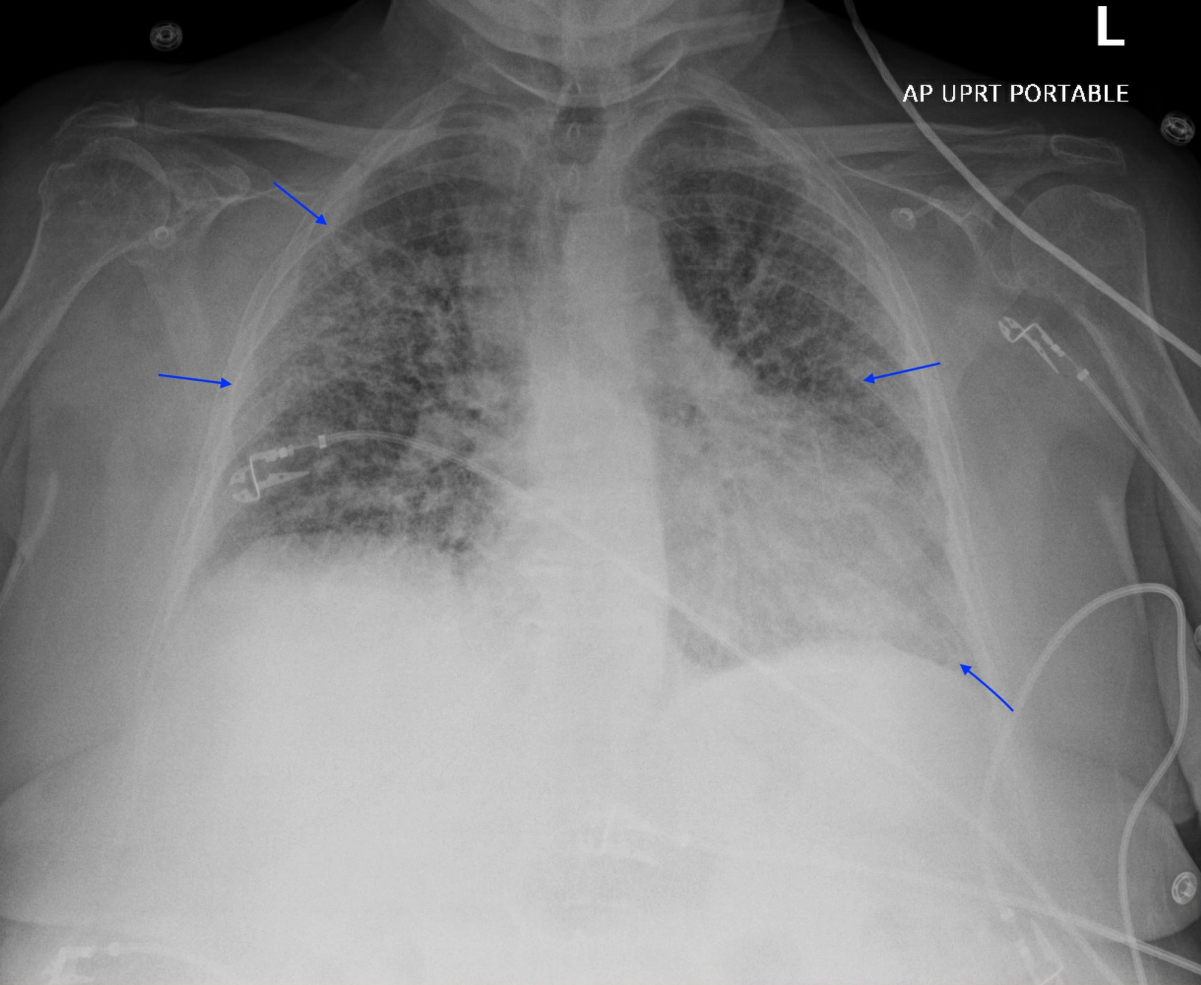
Chest X-Ray Worsening of the bilateral multifocal airspace opacities comparing to prior studies. L: left; AP: anterior-posterior; UPRT: up-right.

**Figure 5:**
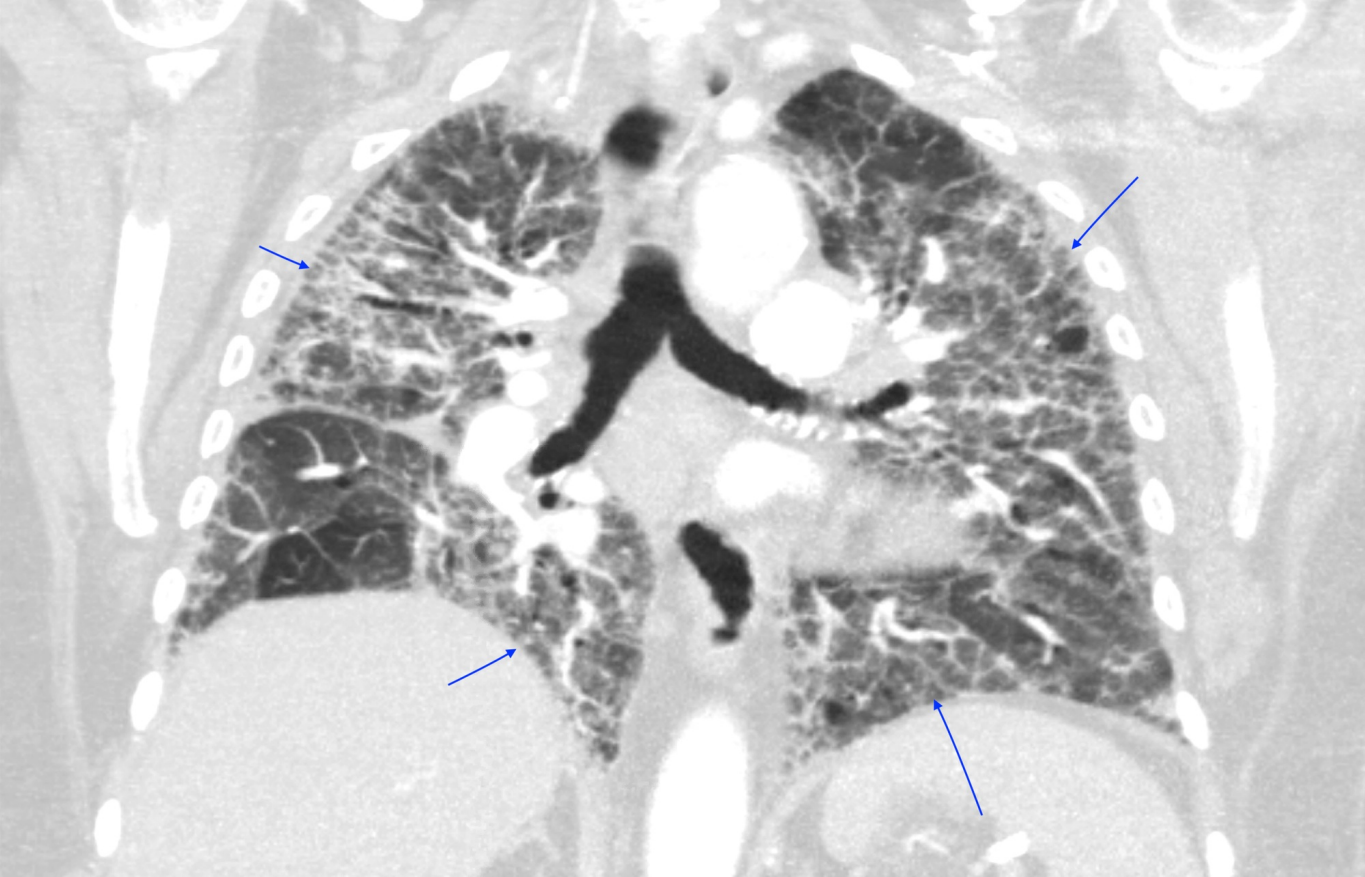
CT Chest (Coronal View) Progression of bilateral alveolar opacities, interlobular septal thickening, and ground-glass attenuation throughout the lungs without intraluminal defects.

The patient was readmitted to the hospital three days after hospital discharge with the chief complaint of worsening of shortness of breath. Initial vital signs on ED presentation: temperature max 99.0°F, blood pressure 132/63 mmHg, heart rate 92 beats per minute, respiratory rate 24 breaths per minute, and pulse oximetry saturation at 88% on NRB. Physical examination revealed increased work of breathing with decreased bilateral air entry with diffuse rhonchi noted. Initial laboratory findings were significant for: serum sodium 133 mEq/L, cardiac troponin 0.24 ng/mL without electrocardiogram changes, creatinine 0.72 mg/dL, white blood cell count 22.2 K/uL, hemoglobin 13.9 g/dL, absolute lymphocyte count 0.6 K/uL, d-dimer plasma 424 ng/mL DDU, cardiac BNP 50 pg/ml, and negative Abbott RealTime SARS-CoV-2 assay. Arterial blood gas (ABG) revealed pH 7.50, the partial pressure of carbon dioxide (_P_CO_2_) 35.1 mmHg, the partial pressure of oxygen (pO_2_) 52.7 mmHg, bicarbonate 26.6 mmol/L, lactic acid 2.09 mmol/L. CXR showed worsening of the bilateral multifocal airspace opacities comparing to prior studies (Figure 4). CT of the chest with contrast revealed progression of bilateral alveolar opacities, interlobular septal thickening, and ground-glass attenuation throughout the lungs without intraluminal defects (Figure 5). The review of systems was otherwise negative. IV methylprednisolone 40 mg t.i.d. was reinstated along with Bactrim for PCP prophylaxis. Pan-cultures were also rechecked.

On day 2 of hospitalization, the patient had worsening of gas exchange and became dyspneic with mild activities accompanied by brisk oxygen desaturation. She was subsequently upgraded to the MICU with high flow nasal cannula (HFNC) supplemental oxygen support and ultimately required bilevel positive airway pressure (BiPAP) ventilation. Flexible bronchoscopy with bronchoalveolar lavage (BAL) revealed scant secretions, normal bronchial tree anatomy, and pink fluids that were inconsistent with diffuse alveolar hemorrhage (DAH). Fungal and routine cultures, cytology were sent from BAL fluids. The endotracheal SARS-COV-2 assay was negative. The transbronchial lung biopsy could not be performed as the patient had been taking clopidogrel. The patient subsequently had worsening hypoxic respiratory failure requiring mechanical ventilator support postprocedure. She was managed with low tidal volume support along with a target plateau pressure <30 cm of water in the setting of an acute respiratory distress syndrome (ARDS) physiology. She continued to receive steroids, diuretics, and Bactrim while monitoring her respiratory status.

**Figure 6:**
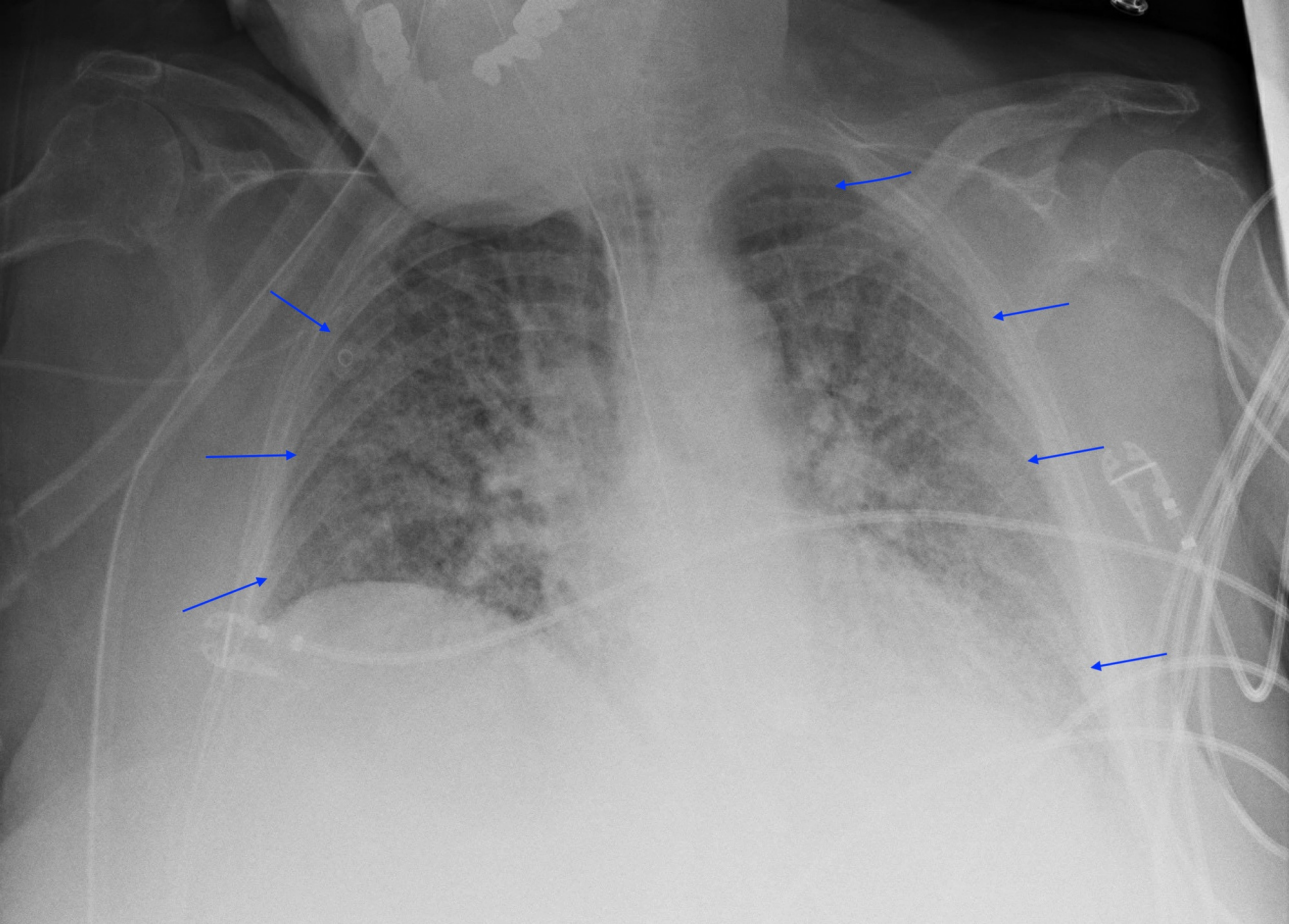
Chest X-Ray Stable diffuse interstitial infiltrates with mild heterogeneous alveolar confluence.

On day 3-12 of hospitalization, the patient remained to be hemodynamically stable and was tolerating spontaneous breathing trials (SBT) while on mechanical ventilator support. Connective tissue serologies along with the autoimmune panel workup were unremarkable. BAL analysis revealed *Rothia* species along with 43% fluid neutrophils, 2% fluid lymphocytes, 52% fluid monocytes, 1% fluid eosinophils, and negative methenamine silver stain. Repeat CXR showed no interval improvement comparing to prior studies (Figure 6). She was subsequently liberated off of invasive mechanical support to BiPAP support successfully after tolerating a few days of SBT. The patient confirmed her wishes to be placed on hospice directed care after making the decision of removing her BiPAP support. She remained to be severely dyspneic, frail, and weak while on HFNC. She unfortunately passed away a few days later. An autopsy was not performed per family wishes.

## Discussion

Hamman-Rich syndrome is often associated with a prodromal illness that lasts around 7-14 days prior to the rapid onset of respiratory failure [4]. Our patient was in her normal state of health until she developed fever, non-productive cough, and dyspnea that failed to respond to outpatient management by her primary care physician thus warranted hospitalization. Initial workup revealed acute hypoxic respiratory failure with findings of bilateral patchy asymmetrical opacities without cardiomegaly or pleural effusions noted on the CXR. Her hypoxic respiratory failure had ARDS physiology based on the Berlin definition due to the acuity of the respiratory symptoms, CXR findings, and the lack of evidence for cardiogenic pulmonary edema based on her clinical impression, echocardiogram results, and cardiac BNP [5]. Furthermore, routine laboratory findings are often useful for excluding Hamman-Rich syndrome as certain connective tissue diseases are associated with interstitial pneumonia that may cause acute exacerbation of respiratory failure [6]. Our patient had normal laboratory findings of serum muscle enzymes, ANA, anti-Jo-1 antibody, anti-CCP, cardiolipin antibodies, and ANCA, thus had a low likelihood of having an underlying connective tissue disease-related interstitial pneumonia. Moreover, a typical chest imaging of ARDS is important when formulating the diagnosis of Hamman-Rich syndrome. In a retrospective radiographic chart review performed by Primack et al. in patients with either biopsy or autopsy-proven Hamman-Rich syndrome, all of the patients had bilateral air-space opacifications found on the CXR along with ground-glass attenuation appearance on the CT scans. These findings suggested that the radiographic findings are similar to those of the ARDS [7]. We were able to perform serial images throughout our patient's hospitalization that revealed continuous worsening of radiographic findings which correlated with her progressive clinical status. In addition, it is also important to rule out infectious causes of respiratory failure with microbiologic investigations from various forms of culture data. However, most patients with Hamman-Rich syndrome have a non-productive cough that makes it difficult to generate sufficient sputum for analysis. Bronchoscopy with BAL would then be a useful tool to obtain samples from the respiratory tract [8]. Our patient had negative culture data to support an alternative infectious process along with a negative methenamine silver stain that likely ruled out PCP pneumonia. Interestingly, diagnosing Hamman-Rich syndrome in the setting of the COVID-19 pandemic increases the complexity of the workup due to similar clinical and radiographic findings. In a retrospective study conducted from three hospitals from the People's Republic of China, certain patients with confirmed COVID-19 diagnosis had CT chest findings of extensive ground-glass opacities and consolidations in both lungs [9]. Our patient's initial diagnosis revolved around a possible atypical presentation of COVID-19 pneumonitis despite an initial negative Abbott RealTime SARS-CoV-2 assay. However, COVID-19 pneumonitis was later eliminated from the differential diagnosis as future COVID-19 testings were negative.

Bronchoscopy with BAL is an important tool used to exclude other diseases with similar clinical presentations. The cellular profile found in Hamman-Rich syndrome is often non-specific. In a retrospective chart review conducted in patients with histologically proven Hamman-Rich syndrome, some of the patients had an increase in neutrophil percentage similar to our patient, although the finding could be non-specific [8]. However, we were able to effectively rule out other pulmonary conditions with similar presentations. Diffuse alveolar hemorrhage (DAH) was eliminated from the differential diagnosis based on the lack of alveolar hemorrhage found during the procedure [10]. Acute eosinophilic pneumonia (AEP) was determined to be unlikely based on the 1% fluid eosinophils noted in the fluids as BAL fluid typically reflects >25 percent of eosinophils in AEP [11]. Furthermore, hypersensitivity pneumonitis (HP) was a compelling alternative diagnosis in this patient due to her occupation as a florist along with similar CT chest findings. In contrast to Hamman-Rich syndrome, HP is often associated with marked lymphocytosis (>20%) found on BAL in the subacute phase [12]. Typical CT chest findings could show centrilobular nodules with ground-glass attenuation with upper lung predominance depending on the disease phase [13, 14]. There were only 2% lymphocytes found in the BAL fluids of our patient along with a lack of centrilobular nodules and upper lobe predominance findings based on the CT scan.

One of the limitations of our case report is the lack of histological evidence. The transbronchial biopsy was not attempted as she was taking clopidogrel in the setting of prior stent deployment. Furthermore, there were discussions of potentially performing a video-assisted or open lung biopsy but later was deemed unnecessary as the patient was medically unstable and later pursued hospice directed care. Hamman-Rich syndrome has the histopathologic appearance of diffuse alveolar damage with phases of acute exudative, organizing proliferative, and fibrosis. The exudative phase develops within first week of the injury that is characterized by intra-alveolar edema, hyaline membrane formation, and interstitial infiltrate of inflammatory cells. Moreover, the proliferative phase occurs one week after the exudative phase that is associated with fibroblastic proliferation throughout the alveolar sac and interstitium. Extensive fibrosis and the organization process is seen histologically in the final phase [3].

## Conclusions

Hamman-Rich syndrome is often a diagnosis of exclusion in the setting of sudden onset of fulminant acute hypoxic respiratory failure with ARDS physiology. Despite histological evidence is important for diagnosis confirmation, the ability to narrow down the differential diagnosis in the proper clinical setting is an essential tool that clinicians should utilize in order to formulate a likely diagnosis and subsequent treatment plan. Using objective data from laboratory findings, bronchoscopy with BAL, and radiographic findings are crucial when making prompt clinical decisions, especially in situations when it is difficult to obtain pathological evidence.
